# A rare case of aggressive uterine leiomyosarcoma: a case report

**DOI:** 10.11604/pamj.2024.49.10.42105

**Published:** 2024-09-09

**Authors:** Subrata Das, Srishti Srivastava, Pragati Srivastava, Nisha Prasad, Madhurima Roy, Inisha Sarkar

**Affiliations:** 1 Department of Obstetrics and Gynaecology, ESI PGI MSR, ESIC Medical College and ESIC Hospital and ODC (EZ), Diamond Harbour Road, Joka, Kolkata, West Bengal, India, Pin-700104

**Keywords:** Uterine leiomyosarcoma, leiomyoma, postmenopausal bleeding, chemotherapy, case report

## Abstract

Uterine leiomyosarcoma is a rare aggressive uterine malignancy that arises from a smooth muscle of the uterus which accounts for 2-5% of all uterine malignancies. Definitive treatment is surgery with a high rate of recurrences. Our patient presented with lower abdominal pain and mass per abdomen which was diagnosed to be uterine leiomyosarcoma. A 56-year-old woman of East Indian origin presented with abdominal pain and a huge rapidly growing suprapubic abdominal mass with an almost monthly doubling. Her CA 125 and Lactate dehydrogenase (LDH) level was elevated and Computed Tomography (CT) scan showed a large irregular-shaped abdominopelvic solid heterogeneously enhanced lesion with focal central hyperdensity and areas of necrosis causing mass effect. A primary cytoreductive surgery was performed and the histopathology report confirmed the diagnosis of uterine leiomyosarcoma. A combination chemotherapy of six cycles was given to prevent recurrence. No recurrence was detected during her more than two years follow-up period. As the cases are rare in nature, screening is impractical. Hence, the diagnosis of uterine leiomyosarcoma is done by histopathologic examination after surgery.

## Introduction

Uterine leiomyosarcoma is a rare aggressive tumour accounting for 2-5% of all uterine malignancies and less than 1% of female genital malignancies [1,2]. The peak age of presentation is the 5^th^ or 6^th^ decade of life [3]. The malignancy originates from smooth muscle cells of the uterus. Prior pelvic radiation and prolonged Tamoxifen intake for breast carcinoma are considered to be risk factors for sarcoma formation [1,4]. The sarcomatous changes of the benign uterine leiomyomas occur in 0.13% to 0.81% of cases. It is a rapidly growing tumour with metastases mostly through the haematogenous route; lymphatic spread can occur rarely. Intermenstrual bleeding and/or rapidly growing uterine mass are the usual presenting symptoms. The incidence of uterine leiomyosarcoma in post-menopausal women is 1-2%. Total abdominal hysterectomy (along with tumour) with bilateral salpingo-oophorectomy is considered optimum treatment, the pelvic lymphadenectomy with para-aortic lymph node evaluation is needed rarely. Surgery is the main way of treatment and prognosis depends on the extent of disease at the time of surgery. A patient with extra pelvic disease at the time of diagnosis will not benefit from surgery. Postoperative adjuvant chemotherapy and/or radiotherapy is used to prevent recurrence [3]. We report a case of this rare condition which presented in a post-menopausal eastern Indian woman, who survived more than 25 months after surgery with no evidence of metastasis.

## Patient and observation

**Patient information and observation:** A 56-year-old post-menopausal woman of east Indian origin with parity 3 and three living children, reported to our out-patient gynaecology clinic with complaints of abdominal pain and with a rapidly growing mass for the past three months. She is a known case of hypertension and hypothyroidism well controlled on medication, with no significant family history of malignancy.

**Clinical findings:** she gave a history of lower abdominal pain which was duly aching in nature. She gave no history of post-menopausal bleeding. Her family history was also not significant. On examination, pallor was present and the patient´s vital signs were normal. She was thinly built and poorly nourished. On examination, an abdominopelvic mass with restricted mobility of a thirty-week pregnant uterus size was found and was pulled up with restricted mobility.

**Timeline of current episode:** the patient´s first visit to the outpatient department was on 15/02/2022 and was admitted on the same day for investigations and treatment. Routine blood investigations and MRI of the whole abdomen were done on 17/02/2022 which showed a large irregularly shaped abdominopelvic solid mass likely to be malignancy originating from the uterus. Computed tomography of the thorax done on 18/02/2022 showed no evidence of metastasis. CA 125, LDH were done on 21/02/2022 showing a marginally raised but other ovarian tumour markers i.e. AFP and beta HCG were within normal range. Upper and lower G.I. endoscopy done on 22.02.2022 showed normal findings. Exploratory laparotomy was done on 28.02.2022. The histopathology report dated 15/03/2022 confirmed the diagnosis of uterine leiomyosarcoma. The oncologist suggested a chemotherapy regimen of doxorubicin, Ifosfamide and Mesna for six cycles on 16/03/2022 and that was followed.

**Figure 1:**
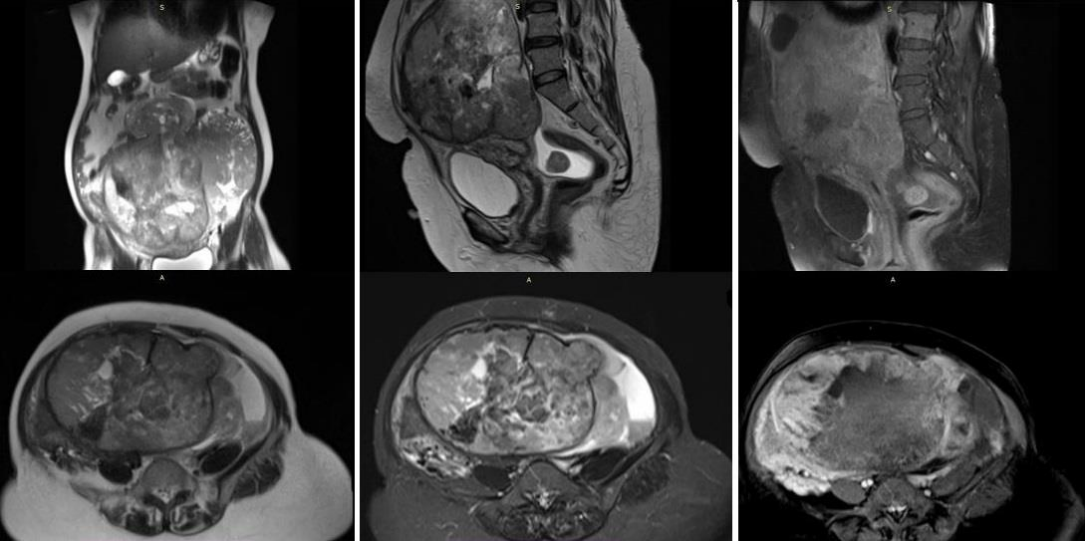
preoperative MRI pictures of the lower abdomen showing the presence of a large abdomino-pelvic tumour originating from pelvic cavity

**Figure 2:**
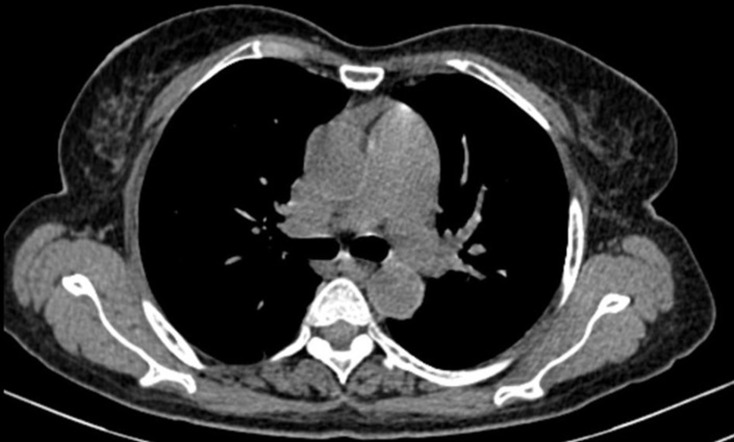
preoperative transverse section of chest computed tomography in lung showing no evidence of metastasis

**Diagnostic assessment:** routine pre-op investigations were normal. Her serum CA 125 level 204.6 U/ml and LDH level 868 U/L were elevated. Magnetic resonance imaging (MRI) studies showed a large irregular-shaped abdominopelvic solid heterogeneously enhanced lesion measuring 16.8cm x 25.4cm x 23cm (approx.) with focal central hyperdensity and areas of necrosis causing a mass-effect neoplasm (Figure 1). Computed tomography of the thorax showed no evidence of metastasis (Figure 2).

**Diagnosis:** differential diagnoses of our case were large uterine leiomyoma, uterine sarcoma and uterine leiomyosarcoma.

**Figure 3:**
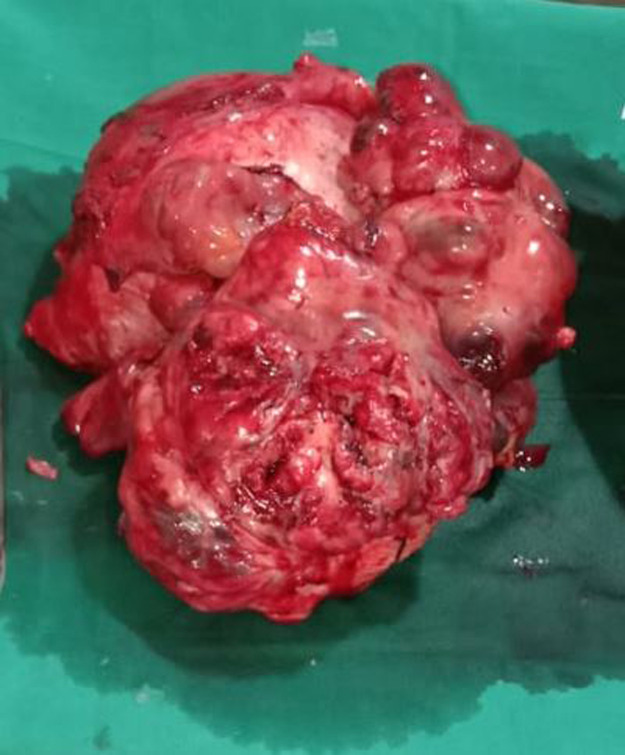
postoperative gross appearance of the tumour

**Figure 4:**
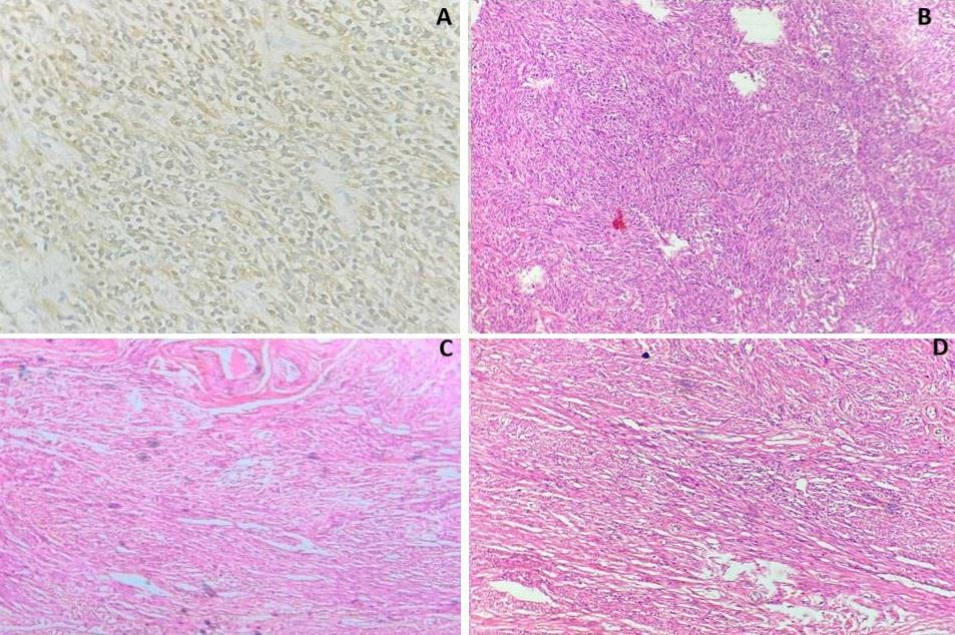
A) immunohistochemistry staining of SMA marker positive (40x); B) (100x, H and E) Showing fascicles of smooth muscle cells exhibiting atypia of Leimyomattous components; C) (100x 10, H and E) showing cells having enlarged irregular nuclei with dispersed chromatin, prominent nucleoli, and having prominent mitotic activity; D) (100x, H and E) showing sarcomatous component of Leiomyosarcoma showing spindle-shaped tumour cells arranged in interlacing bundles of spindle cells with elongated hyperchromatic nuclei and nuclear pleomorphism

**Figure 5:**
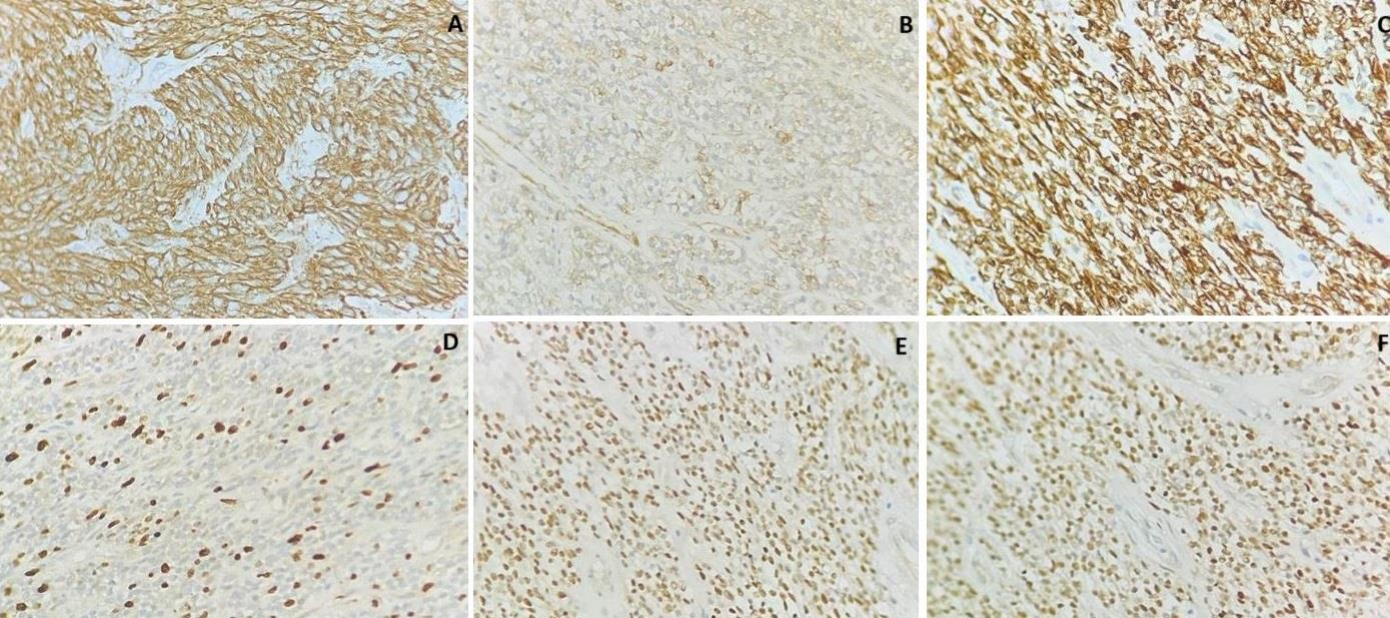
postoperative pathological immunohistochemistry (IHC) staining of six markers, (40x): A) caldesmon positive; B) CD 10 receptor negative; C) desmin positive; D) Ki 67 positive; E) estrogen receptor (ER) positive; and F) progesterone receptor (PR) positive

**Therapeutic intervention:** exploratory laparotomy was performed with simple total abdominal hysterectomy and bilateral salpingo-oophorectomy. Intraoperatively, 200 ml of serous ascitic fluid was noted. The uterus was enlarged around 30 weeks size. The left ovary was normal. The left fallopian tube was stretched over the mass. A multiloculated 30x20x20cm mass was infiltrating and stretching the uterus. Mass was wrapped by a sigmoid colon and its mesentery, omentum and pelvic peritoneum. En bloc removal of the uterus with ovarian mass, gastric arcade sparing omentectomy, bilateral pelvic lymphadenectomy and para-aortic lymph node sampling were done (Figure 3). Hematoxylin and eosin (H&E) staining showed microscopically atypical spindle cells and revealed interlacing fascicles without necrosis consistent with uterine leiomyosarcoma of FIGO stage 2B (Figure 4). Immunohistochemistry showed diffused reactivities of tumour cells and positivity for Estrogen Receptor (ER), Progesterone receptor (PR), Caldesmon, Desmin and SMA receptors. There is patchy reactivity in tumour cells for CD10, Cyclin D1 and negative staining for CD117(C-Kit), Chromogranin A, HMB45 and Inhibin (Figure 4, Figure 5). Histopathology confirmed the excised mass was spindle cell neoplasm which was most consistent with uterine leiomyosarcoma. Postoperatively, the patient underwent all routine investigations and was given six cycles of chemotherapy in the form of: injection doxorubicin 20 mg/m^2^/day IV on days 1-3 plus; injection ifosfamide 1,500 mg/m^2^/day IV over 3 hours on days 1-4 plus and injection mesna 225 mg/m^2^ IV over 1 hour before injection ifosfamide and at 4 and 8 hours after injection ifosfamide and each cycle was repeated every 21 days.

**Follow-up:** the echocardiography and positron emission tomography (PET) Scan was done after the completion of her sixth chemotherapy cycle which was found to be within normal limits. Post-chemotherapy, the woman was on regular follow-up for 25 months, there was no evidence of recurrence, but thereafter woman was lost to follow-up. Intervention adherence was satisfactory and no unanticipated events were reported.

**Patient perspective:** I was very anxious about the growing swelling in my abdomen. I feel relief after discharge. Very grateful for the timely treatment that I received.

**Informed consent:** written informed consent was obtained from the patient for publication of this case report and any accompanying images.

## Discussion

Uterine leiomyosarcoma is a rare form of aggressive uterine malignancy and considered to originate from embryonic mesenchymal cells [5]. Most common presentation of leiomyosarcoma are post-menopausal bleeding per vagina, rapidly growing suprapubic mass and pain abdomen [6,7] or pressure symptoms, but in our patient, the only presentation was rapidly growing suprapubic mass extending up to upper abdomen.

Serum CA 125 and lactate dehydrogenase (LDH) are used as a tumour marker for early diagnosis of leiomyosarcoma but both of the markers have poor sensitivity and specificities [5]. Uterine leiomyoma, sometimes co-exist with leiomyosarcoma. Different types of degeneration occur in uterine leiomyoma e.g. hyaline degeneration, cystic degeneration and also sarcomatous degeneration. It is considered that 1 in 800 fibroids in the uterus, is a Leiomyosarcoma [8]. It is seen that, the incidence of unexpected detection of uterine sarcoma in 1 in 352 laparoscopic hysterectomy specimens and 0.5% of hysterectomy specimens which was assumed to be uterine fibroid [7]. Pre-operative ultrasonography or CT scan has poor sensitivity to distinguish sarcoma from Uterine leiomyoma. It is very difficult to diagnose in a frozen section also. Only an MRI guided biopsy has the capacity of pre-operative diagnosis of Leiomyosarcoma with good sensitivity [4]. MRI appearance of leiomyosarcoma is variable, it appears as infiltrating large myometrial heterogenous mass with low signal intensity on T1-weighted images, with irregular and ill-defined margins.

It is a very aggressive tumour with a high rate of local recurrence and the most common site of distant metastasis is the lung. Isolated recurrence, treated with excision. Prognosis depends on the size of the tumour more than 5cm, mitotic rate, heterologous elements, necrosis, myometrial invasion, sarcomatous overgrowth and lymphovascular space invasion and stage during diagnosis [7]. Chances of distant spread increase in morcellated specimens of those undergone laparoscopic hysterectomy. For such cases, removal of the uterine specimen through the vagina should be considered to prevent distant spread. Survival of the patient depends on complete resection of the tumour during initial surgery [8], combination chemotherapy has a limited role in the survival of the patient [9]. The tumour is very aggressive and high risk of local recurrence. In our patient, though the tumour mass was very big, we achieved complete resection of the tumour during the time of surgery. There were no documented metastatic spreads. We used the six-cycle combination chemotherapy with doxorubicin, Ifosfamide and Mesna to prevent recurrence of the tumour. During our 25^th^ month follow-up, the patient remained disease free and she was lost to follow-up thereafter.

## Conclusion

A 56-year-old post-menopausal woman presented with a rapidly growing lump in the abdomen and after investigation and surgical intervention, was diagnosed to be a rare case of leiomyosarcoma. Patients are required to be thoroughly investigated if they are within the post-menopausal age and present with rapidly growing uterine mass and/or bleeding per vagina to rule out leiomyosarcoma. So high index of suspicions is needed to diagnose and treat such patients timely. Leiomyosarcoma is a very aggressive tumour but no screening method is applicable in such patients as it is very rare and also for non-availabilities of sensitive and specific tumour markers.
